# The Impact of Dust Particles on the Function of Screen‐Printed Triple‐Mesoscopic Perovskite Solar Cells

**DOI:** 10.1002/smll.73299

**Published:** 2026-04-07

**Authors:** Kathryn Lacey, Sarah‐Jane Dunlop‐Potts, Carys Worsley, Rodrigo Garcia‐Rodriguez, Tom Dunlop, Declan Hughes, Krishna Seunarine, Matthew Davies, Trystan Watson

**Affiliations:** ^1^ SPECIFIC IKC Faculty of Science & Engineering Swansea University Swansea UK; ^2^ AIM Faculty of Science & Engineering Swansea University Swansea UK; ^3^ School of Chemistry and Physics University of KwaZulu‐Natal Durban South Africa

**Keywords:** carbon, cleanroom, dust, mesoporous, perovskite, screen printing

## Abstract

Screen‐printed mesoporous carbon perovskite solar cells (m‐CPSCs) offer a low‐cost manufacturing approach that could significantly reduce upscaling costs, providing an accessible pathway to green energy production opening new opportunities in countries where this is currently cost‐prohibitive. Clean rooms represent a very expensive investment and overhead, presenting a significant barrier to reducing production cost. Despite this, very little research has been carried out to determine whether a clean room is essential to PSC manufacture. This study examines how organic dust particle contaminants (1–10 µm) impact screen‐printed m‐CPSCs. Unexpectedly, cells with contamination between layers showed no significant difference in PCE compared to pristine controls. Dust in pastes affected print quality more severely, negatively impacting the quality of subsequent layers and leading to inconsistencies in print quality, compounding problems further along in the manufacturing process if screens and pastes are not kept free from such contamination. These findings suggest that screen‐printed solar cells can be produced outside of clean rooms if high cleanliness standards are maintained during storage and print runs. This outcome is significant for low‐cost PSC printing, indicating that it may be possible to produce high performing modules in industrial setups without expensive clean room installation and management.

## Graphical abstract

Screen‐printed mesoporous carbon perovskite solar cells offer an accessible route to scalable green energy. This study investigates the impact of dust particle contamination during the manufacture of this type of solar cell. Results suggest clean rooms may be unnecessary if strict cleanliness is maintained during storage and printing processes in industrial production environments.

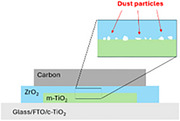

## 1 Introduction

Perovskite solar cells (PSCs) are a promising generation of photovoltaic devices that offer many advantages over other types of solar cells. Relatively resilient to crystallographic and electronic defects, and amenable to solution‐based processing, PSCs have the potential to be low‐cost and straightforward to produce, and many now exceed 25% efficiency [1].

PSCs can be constructed with varying architectures using many deposition techniques. One potential deposition method is screen printing, a well‐established method already used for manufacturing technologies such as circuit boards and sensors using functional inks. Of the many potential manufacturing techniques for PSCs, screen printing is a particularly accessible method, utilizing simple equipment that is easily scaled [2, 3].

Herein, we investigate m‐CPSCs, a device architecture based on three screen‐printed mesoscopic layers: a titanium dioxide (TiO_2_) electron transport layer (ETL), a zirconium dioxide (ZrO_2_) insulating layer, and a carbon top contact. Each layer is annealed separately to remove ink solvents and binders, leaving behind a mesoporous scaffold throughout which the perovskite solution can be infiltrated.

PCEs reported for m‐CPSCs have exceeded 20% in recent years [4, 5, 6, 7]. They exhibit superior device stability and lifetime compared to many planar PSC architectures [8, 9, 10] and are showing promise with larger‐scale modules in outdoor environments [11, 12, 13]. These attributes make m‐CPSCs a potential frontrunner to PSC commercialisation. Methylammonium lead iodide (MAPbI_3_) with added 5‐AVAI is currently one of the most reproducible and stable perovskites in m‐CPSCs, having passed rigorous IEC stability tests [10].

Despite this progress, translation to scalable manufacturing remains challenging. As with many thin‐film electronic technologies, fabrication is typically carried out in cleanroom environments to minimize dust contamination of printed films. Such contamination is shown to significantly lower device performance in silicon solar cells [14, 15].

As m‐CPSCs only require relatively low‐cost equipment, obtaining and maintaining a clean room represents an expensive initial and ongoing investment, offsetting low‐cost production methods, potentially making m‐CPSCs less accessible [16] and representing a barrier to industrial‐level upscaling in developing countries.

Clean rooms are often used for research‐level and high‐performance m‐CPSCs as it is often assumed that printing outside of an environment where there are strong controls on atmospheric dust particles may result in poorer quality and lower performing devices [14, 15]. However, no data currently exists on whether m‐CPSCs are susceptible to these limitations. If the screen‐printing process can be reliably done outside of a clean room environment without impacting the device performance of the cells, this has significant advantages in terms of setup costs.

There are several ways in which particle contamination could impact m‐CPSCs. Interfacial recombinations are the dominant location of recombination losses in PSCs [17]. Due to the requirement to allow films to dry and anneal after printing, print interfaces are vulnerable to dust contamination, reducing interfacial connectivity or blocking perovskite infiltration, effectively reducing active area and contact with the TiO_2_ layer [18, 19]. Particle contamination of printing inks may also have a detrimental impact, affecting ink rheology and producing thinner, rougher prints with higher potential for pinholes.

Our previous study, analyzing the impact of dust particle contamination in planar PSCs, noted only very minimal impacts on the performance of devices, even at high levels of contamination, suggesting that perovskite films have a good resistance to physical contaminants such as these [20]. However, in m‐CPSCs, as the perovskite precursor is infiltrated throughout the mesoporous stack, there may be different mechanisms to consider. For example, particular carbon flake orientations in the carbon top contact have previously been found to cause infiltration defects, suggesting that it is possible for large particles to interrupt infiltration and produce inactive areas [21].

In organic solar cells, dust particles have been found to locally reduce the charge extraction rate [22]. While these effects appear to have limited impact in planar PSCs, the presence of mesoporous layers introduces additional interfaces and transport pathways that may increase sensitivity to particulate contamination.

This study quantitatively investigates how dust particles between the printed layers of m‐CPSCs impact performance, as well as examining how paste dust contamination affects the printing process and resultant layer quality. This enables an assessment of whether m‐CPSCs can viably be made outside of a clean room environment. Such analysis will help inform research labs looking to manufacture m‐CPSCs as well as potential large‐scale commercial initiatives.

## 2 Results and Discussion

### 2.1 Experimental Methods

As each mesoporous layer is individually printed and annealed, there is an opportunity for contamination at each interface. Airborne particles can easily settle on prints either as they slump before annealing or while drying. The first part of this study, therefore, simulates airborne dust particle contamination of fresh prints, comparing clean devices with those exposed to moderate dust level (<500 µg m^−3^), or high dust level (>1000 µg m^−3^) contamination environments at each printed mesoporous interface. This experimental setup was first introduced by us in a similar study conducted on planar PSCs [20].

To achieve dust contamination in a controlled environment, fresh prints were placed in an enclosed ‘dust box’ prior to annealing, wherein industrial test dust was circulated to produce controlled contamination conditions. The dust box is a specially designed enclosure where dust can be circulated via a short blast of compressed air or nitrogen, with the samples sat on a platform above this. Dust circulates around and onto the samples undisturbed by other air circulation or contamination, measured as it settles by a small particle counter recording data every second.

The type of test dust used in this study was chosen to consider the physical presence of a typical organic dust particle common to many environments, eliminating the additional complexities of dust particles with other characteristics such as conductivity. Details on the size, morphology, and chemistry characterization of the industrial test dust versus samples from various labs are included in Figure S1.

Further information on the dust box setup and the drop‐out rate of dust particles in the dust box compared to real‐world dust settling rates is detailed in Figure S2. This analysis was also previously carried out in our previous paper on planar perovskite solar cells [20].

Dusty and control devices were printed at the same time; prints requiring dust contamination were placed into the box, and dust circulated. Clean control prints were protected under a cover during this time before concurrent annealing of both sets of prints on a hot plate in a fume hood.

Following dust deposition, the rest of the device was printed as normal. Dust was deposited for sets of devices in the following categories: before the TiO_2_ print, onto the TiO_2_ print (before annealing), and onto the ZrO_2_ print, producing four sets of devices, including control batches.

ISO clean room classifications require clean rooms to have limits on particle sizes between categories ≥0.1 µm (ISO class 1–6) and up to ≥5.0 µm (ISO class 6–8). Dust particle sizes required for this study need to cover all environments currently used in PSC manufacture, ranging from no particulate controls to a class 6 clean room, to assess the level of particle control that is required. Therefore, a test dust with a 1–10 µm particle size range was selected. The test dust is comprised of an organic cotton material which is comparable in shape, size, and composition to much of the dust present in the lab.

**FIGURE 1:**
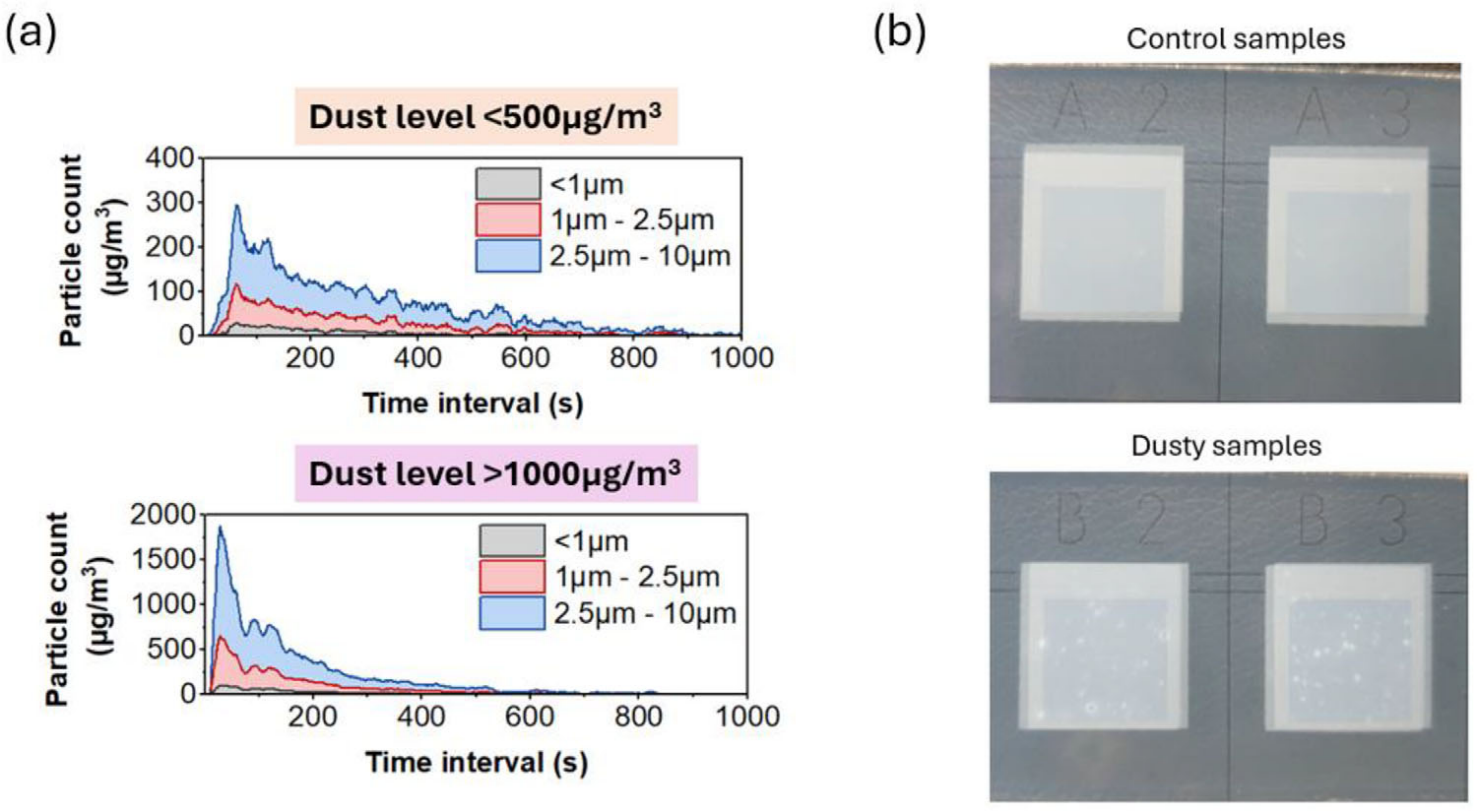
Experiment testing: (a) Comparison of data output from dust box showing results from moderate dust circulation <500 µg m^−3^ versus high level of dust circulation >1000 µg m^−3^; (b) photographs showing the difference between clean and dusty samples with dust at TiO_2_ layer and the ZrO_2_ print over top.

Figure 1a shows examples of data output of particle quantity segregated by size ranges <1, 1–2.5, and 2.5–10 µm at two different dust circulation levels: <500 and >1000 µg m^3^. These two levels of dust circulation are used on samples throughout this study. Once deposited, the dust on the fresh print is clearly visible, as seen in the photographs in Figure 1b, which show dust deposited at the TiO_2_/ZrO_2_ interface in comparison to clean control samples. SEM and EDS analysis post‐anneal of the TiO_2_ layer confirmed that the dust remained in‐situ and unchanged despite the high temperature annealing process (Figure S3).

### 2.2 Interfacial Dust Contamination

**FIGURE 2:**
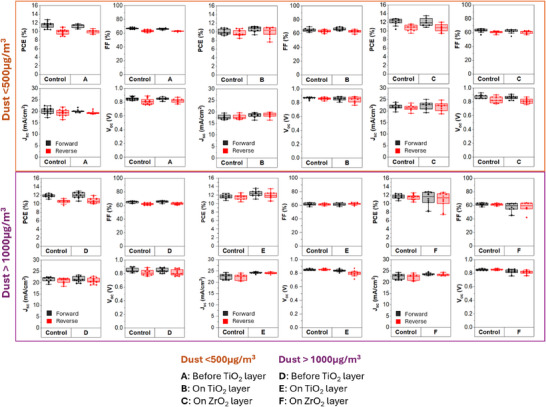
JV data comparison of power conversion efficiency (PCE), fill factor (FF), J_sc_ and V_oc_ of devices with dust located at each print interface, comparing moderate (<500 µg m^−3^) and high (>1000 µg m^−3^) dust contamination levels. Sample size range is noted as *n* = 6–12 each for control samples and dusty samples. This is further indicated by points on the boxplot.

Devices were made and tested in batches to characterize and compare clean controls alongside those with dust deposited at different interfaces. Each set of devices was printed separately and compared to control devices from the same batch. Figure 2 shows the JV data from devices with moderate and high levels of dust at each printed layer. JV curves from hero devices in each batch (control device vs. dusty) are included (Figure S4).

It is clear from this data that interfacial dust does not significantly impact PCE regardless of its quantity or position in the stack. There was no significant difference between average PCE, FF, J_sc,_ or V_oc_ values outside of normal batch variance. This was unexpected given the known sensitivities of other types of solar cells to contamination, and that the thickness and uniformity of printed mesoporous layers is known to be critical, with thin areas driving unwanted recombination and rough prints impacting the quality of the carbon top electrode [23]. This indicates that m‐CPSCs may be more resilient to interfacial dust contamination than expected.

However, although average JV parameters did not significantly change, reproducibility was reduced with interfacial contamination. This was driven by increased J_sc_ variance in devices exposed to moderate contamination, and by FF in those exposed to high contamination. Interestingly, in many cases, reproducibility was increasingly impacted at each interface, with the largest variance with ZrO_2_ contamination at both dust levels.

A closer look at the JV curves of hero devices (Figure S4) from each batch shows that there is a very small drop in V_OC_ in nearly all dusty samples compared to the controls as well, with a contribution of increased shunts apparent with high levels of dust contamination at the ZrO_2_ layer.

These differences may be resultant from variations in print quality or perovskite infiltration caused by dust particles. To investigate the reasons for reduced reproducibility, we considered how dust particle contaminants may influence local variation in print quality, perovskite infiltration or crystal quality. A variety of techniques were employed to look at the quality of the device manufactured in clean versus dusty environments. Dark‐field optical images were taken looking through the glass/ITO to view the printed mesoporous TiO_2_ layer.

**FIGURE 3:**
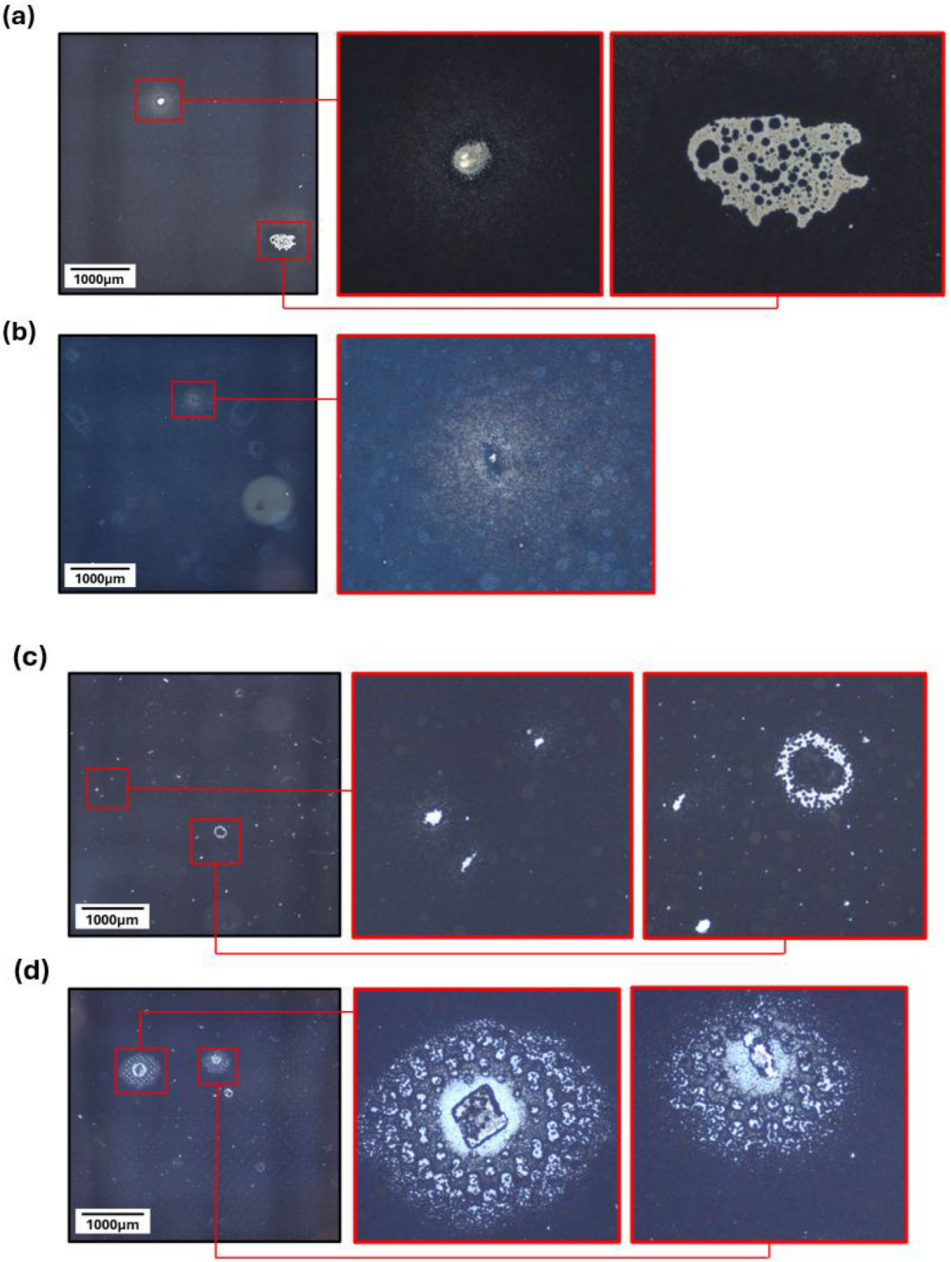
Optical images of a variety of defects in the TiO_2_ layer in dusty devices: (a) particle defect and area of missing TiO_2_; (b) small particle with wide area of mesh marking; (c) various small areas of missing TiO_2_; (d) large particles with significant areas of mesh marking and missing TiO_2_.

Figure 3 shows optical images of several different particle defect types in devices with moderate levels of dust above (Figure 3a,b) and below (Figure 3c,d) the TiO_2_ print. These defects were not observed in clean samples.

A variety of defects were observed in samples with dust contaminants below and above the m‐TiO_2_. In general, deposited dust particles remain at the defect site, causing thinning or absence of the surrounding print or an area of significant mesh marking. Mesh marks are typical features in screen prints [19], caused by where the threads of the mesh cross over each other and can be detrimental to the function of a device by changing the roughness of the surfaces. They can be minimized with correct print settings but also exacerbated in situations such as this, where there is a change in the print surface [19].

The largest particles cause a ring of missing m‐TiO_2_ and significantly mesh‐marked and thinning print around them, as can be seen in Figure 3d, where a 50 µm particle has caused a thinned area of 500 µm diameter, and a 15 µm particle has caused a 300 µm diameter thinned area. Mesh marks are areas in the printing screen mesh where a small peak can be formed in the print at a join in the grid of the mesh. These are a common feature in screen prints and can be mitigated by the mechanics of the print (i.e., pressure, speed) and the properties of the paste (i.e., viscosity).

Mesoporous TiO_2_ mesh marking and thinning were observed in both sample sets, indicating that dust contaminants below and above a given layer can both impact its thickness. Clearly, the cause of layer thinning in each case must be different. Where TiO_2_ has been printed onto an underlying dust particle, the particle likely prevents the mesh fully contacting the surrounding substrate during printing. Significant thinning of the surrounding TiO_2_ can occur in this way, even in the case of very small particles: Figure 3b shows a small particle that has caused TiO_2_ thinning over a large distance. Where thinning has occurred due to dust atop the TiO_2_, this may be a consequence of the particle mass disrupting the underlying film or improper paste settling due to incompatible surface energies between the dust particle and the printing ink. In the second instance, the extent of thinning in the underlying print may therefore be related to the chemistry and surface energy of the contaminant particle.

**FIGURE 4:**
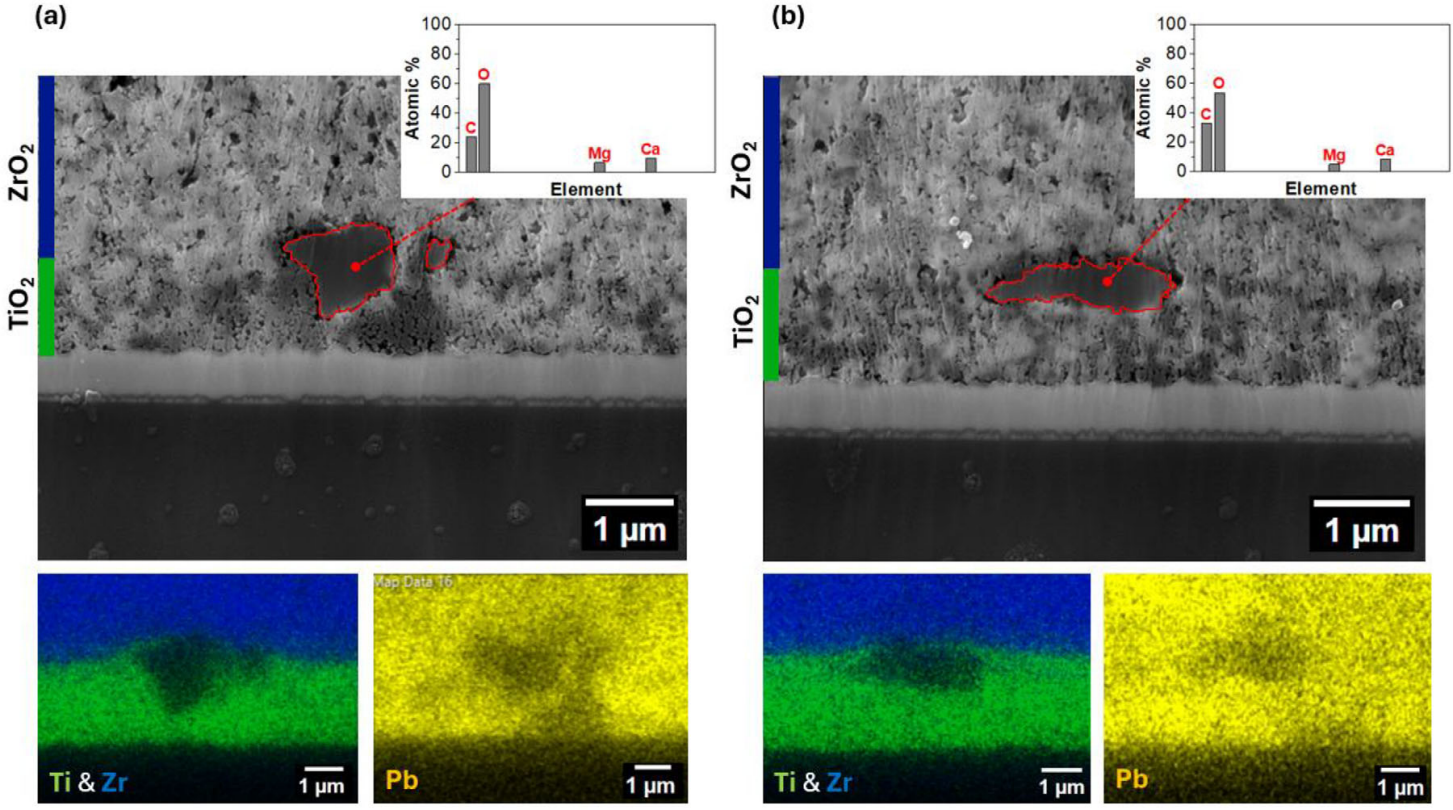
SEM images with EDS point and map analysis of dust particles at printed mesoporous TiO_2_ layer: (a) two dust particles showing disruption to perovskite infiltration; (b) a dust particle showing no interruption to perovskite infiltration.

It is known that print defects such as these can affect the infiltration of perovskite into the mesoporous stack [19]. For insight into how dust particles deposited atop mesoporous layers affect perovskite infiltration and how such contaminants affect the underlying print, cross‐section SEM images were obtained of a device with dust at the TiO_2_ layer (Figure 4). Dust particles were located as expected in these devices between the mesoporous TiO_2_ and ZrO_2_ layers, and positively identified with EDS, which confirmed that the particle chemistry matched that of the test dust.

Two adjacent particles are visible in the cross‐section in Figure 4a, with one measuring approximately 1 µm and the other at around 250 nm. The mesoporous TiO_2_ below these particles appears darker under SEM, suggestive of poor perovskite infiltration in these areas [24]. This is confirmed by the Pb EDS mapping, which shows a much lower Pb signal in the TiO_2_ underneath the dust particles.

Interestingly, not all dust contaminants prevented infiltration. Figure 4b shows a cross‐section of one dust particle around 2 µm across, which did not show an accompanying infiltration defect, establishing that it is possible for the perovskite to infiltrate under and around contaminant particles. Many factors may play a role in impacting perovskite infiltration, including the size or shape of a dust particle, its orientation, and its proximity to other dust particles. It could be hypothesized that a flatter dust particle might inhibit infiltration more by blocking a wider area, although this is not necessarily the case with the smaller particle in Figure 4b, where there is still a good layer of TiO_2_ underneath the flatter particle for the perovskite to infiltrate sideways through. Conversely, the particles in Figure 4a may reveal more about how small particles may impact infiltration as a rounder particle disrupts the TiO_2_ underneath more, creating a thinner layer. The smallest particle next to this also expands the shadowed area in the TiO_2_ layer, leading to more disruption in infiltration.

It can therefore be concluded that small particles do not always negatively affect perovskite infiltration. This may explain the relatively small impact of interlayer dust contamination on overall device performance.

Figure 3 shows that particles can influence print quality directly around the contaminant. Although neither of the surrounding TiO_2_ and ZrO_2_ prints developed pinholes, the TiO_2_ layer was thinner at each particle location. It was posited that contaminants may also likely impact the quality of subsequent prints over top of these inconsistent areas. Controlling layer thickness and topology is critical in all layers, as increased roughness in underlying TiO_2_ or ZrO_2_ has previously been shown to impact infiltration and affect carbon layer conductivity by increasing mesh marking and reducing print coherence [23]. This could explain the lower reproducibility of interlayer contaminated samples.

To examine how interface contamination at each layer can impact subsequent prints, samples were prepared with dust contamination before the mesoporous TiO_2_, after the mesoporous TiO_2_, or after the ZrO_2_. Topography analyses of every printed layer in each sample were obtained using white light interferometry (WLI) to visualize any changes in surface profile at the dust‐contaminated layer and in the subsequent prints atop the contaminants. A corresponding clean control stack was prepared in each case for comparison.

**FIGURE 5:**
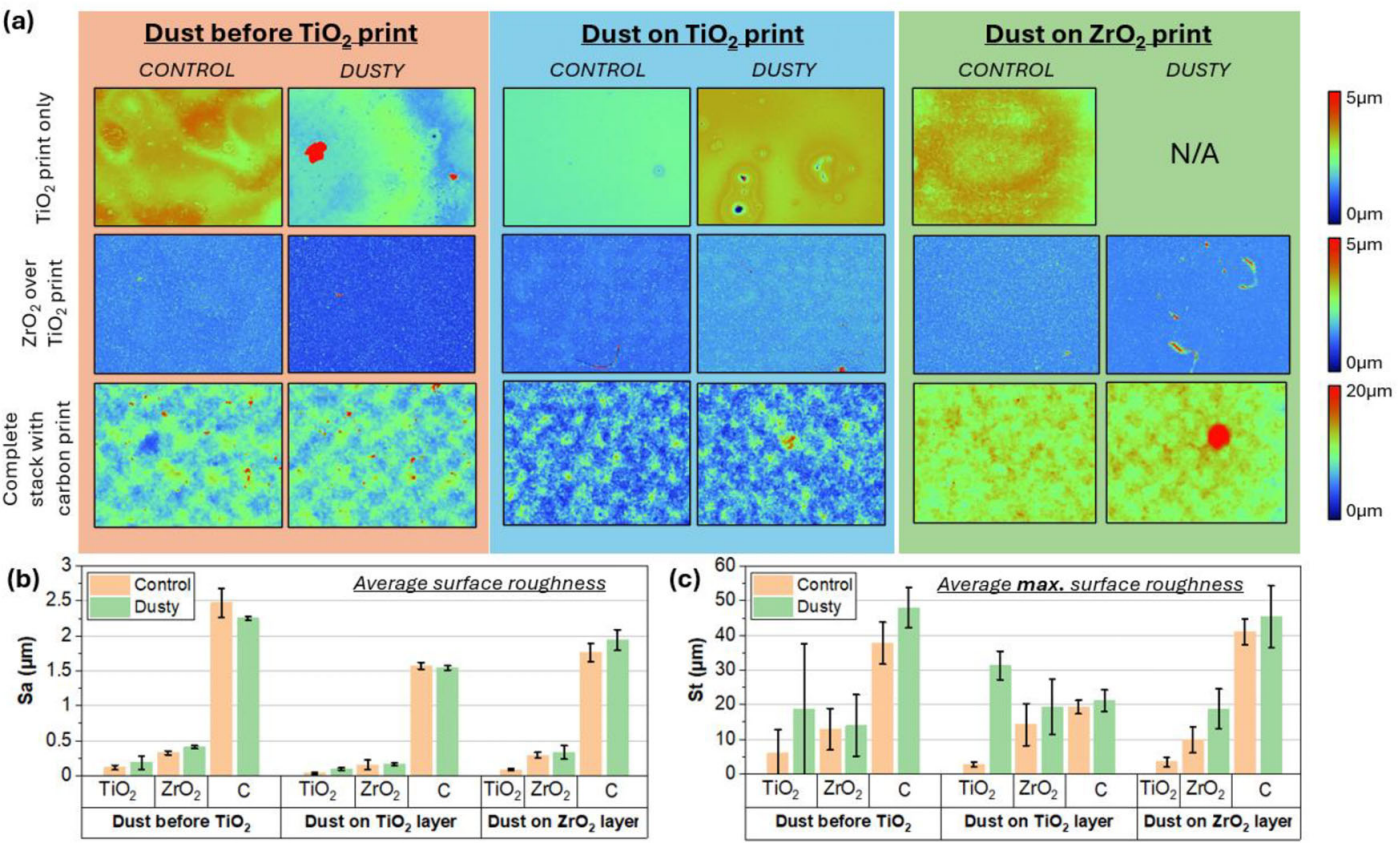
White light interferometry results of control versus dusty prints: (a) on each printed layer with dust deposited before TiO_2_, onTiO_2_ with ZrO_2_ printed over top and on ZrO_2_ with carbon printed over top; (b) comparison of average surface roughness (S_a_) for each printed layer; (c) comparison of average maximum surface roughness (S_t_) for each printed layer.

Figure 5a shows the topographic profiles of control and dusty samples for each sample type. For clarity, the impact of dust at each interface is discussed separately below. Quantitative values for average surface roughness (Sa) (Figure 5b) and average maximum surface roughness (St) (Figure 5c) were also compared.

Rougher prints indicate a lower quality, inconsistent print: a higher incidence of mesh marking (characteristic spaced peaks and troughs left by the mesh on the printed film) and reduced coherence of a print being linked with a lower functioning device [25] at the TiO_2_ layer.

#### 2.2.1 Dust Before TiO_2_

Samples with dust before the TiO_2_ print showed a significant increase in both S_a_ and S_t_ compared to controls, with a range of particles present, including several anomalies of over 40 µm in height. Apart from these large peak defects, the rest of the surrounding print was relatively similar between dusty and control samples, with similar print quality in terms of texture, waviness, and pinhole quantity. Large anomalies on the TiO_2_ print resulted in slightly rougher ZrO_2_ prints than for the clean controls.

#### 2.2.2 Dust on TiO_2_

Both the TiO_2_ and subsequent ZrO_2_ print show a notable increase in both roughness values with dust contaminants atop the TiO_2_ surface. The carbon printed over top of the ZrO_2_ for these samples showed generally very little variance in S_a_ and S_t,_ suggesting that the inherent roughness and thickness of the carbon layer tend to minimize the effect of any increased roughness at previous prints.

#### 2.2.3 Dust on ZrO_2_

The TiO_2_ print for this batch was a control sample only, as dust was not deposited until the ZrO_2_ print. Aside from the presence of dust, the print itself was relatively homogeneous, as was the ZrO_2_ print in the control samples. Dust landing on the wet film did not appear to result in the same thinning as was seen in the TiO_2_ print, perhaps due to the higher viscosity of the ZrO_2_ paste. Although the dusty ZrO_2_ samples had a far higher S_t_ than the controls, indicating a few peaks where dust particles are located, the S_a_ of both clean and dusty ZrO_2_ were very close.

These results show that dust at any layer can impact TiO_2_ and ZrO_2_ average surface roughness. The most significant impact was with the dust being located before the TiO_2_. This is to be expected due to this being the thinnest layer at around 200 nm.

The effect on all values is minimized by the time the carbon layer is applied, regardless of the contaminated interface, due to the greater thickness and roughness of printed carbon layers. However, it should be noted that even a relatively small increase in carbon roughness can have a big impact on device performance, influencing infiltration and charge extraction (as seen in Figure 3). As the highest variance in carbon print roughness was seen when dust was located at the ZrO_2_ layer, this may be the root cause of the increased PCE variance seen in devices with dust deposited onto the ZrO_2_ print.

The resultant topography of the carbon layer is important as this indicates a lower quality print where there may be thinner areas or agglomerations of the carbon black and graphite particles [26], both of which will have a detrimental impact on the performance of a device.

Further analysis was undertaken to understand the impact on charge behaviors that the presence of interfacial dust particles may have. Photoluminescence (PL) and light‐beam induced current (LBIC) mapping have previously been used to visualize differences in photon emission and charge extraction across the active area of mesoscopic devices at multiple scales [24]. To ascertain the cause of reduced reproducibility and the effect of contamination‐driven print defects seen in the optical images, and to examine any smaller‐scale changes in contaminated devices, spatially resolved steady‐state PL and LBIC were measured across control and dusty devices using the same 4000 × 4000 µm area used to obtain JV data.

**FIGURE 6:**
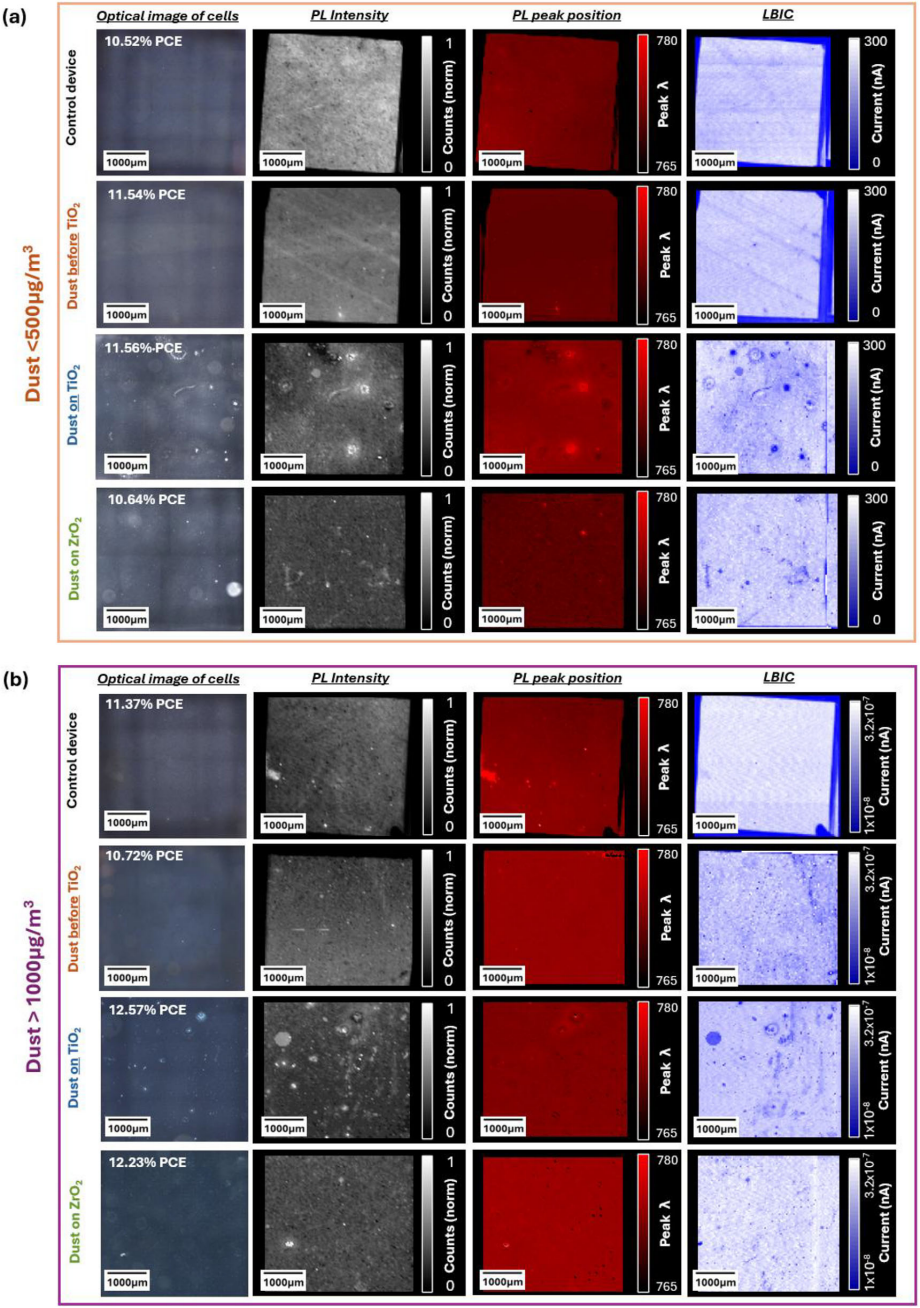
Optical images alongside PL intensity at 775 nm, PL peak position, and LBIC maps; (a) images and maps for moderate dust levels <500 µg m^−3^; (b) images and maps for high dust levels >1000 µg m^−3^.

Figure 6 shows dark‐field optical images alongside PL intensity, PL peak position, and LBIC maps of the same area of complete devices for clean control devices, those with moderate (<500 µg m^−3^) levels of dust, and high (>1000 µg m^−3^) levels of dust exposure at each printed layer. The mapping data provide a spatially resolved comparison between perovskite crystal quality, charge generation, and recombination behavior [17, 27, 28, 29] while the optical images visualize infiltration and print defects in the TiO_2_ caused by dust [19].

Clean control devices from both batches show relatively bright and uniform PL, PP, and LBIC. Optical images show uniformly well‐infiltrated devices with minimal variation. Higher and more homogenous PL intensity matched with higher LBIC indicates reduced non‐radiative recombination and good charge extraction in these devices, and although there are a few local variations in PP, this is also relatively uniform across the control devices. This indicates that these devices have smooth prints, good layer interconnects, and consistent infiltration and crystallization of the perovskite [24].

This contrasts with the dusty samples, which in general show lower PL intensity and much poorer uniformity across PL, PP, and LBIC with a wide variety of localized changes. The pattern of differences generally corresponds closely across all luminescence maps for each sample, matching the same defects seen in the optical images. Clearly, the presence of dust particles at interfaces can affect charge behavior, and this varies depending on the layer in which they are present.

**FIGURE 7:**
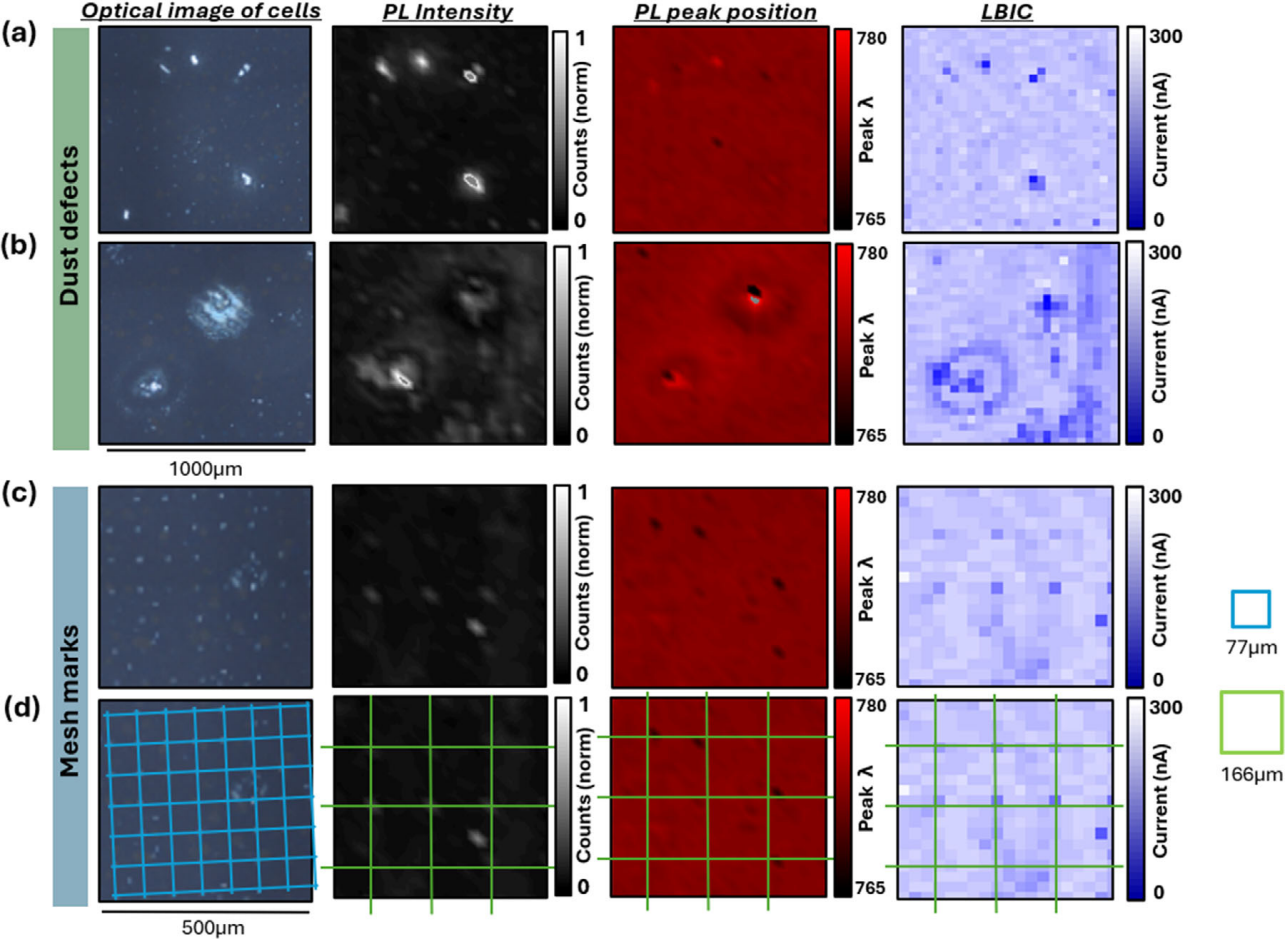
Examples of defects found in PL, PP, and LBIC maps. Examples of defects likely caused by dust contamination: (a) smaller defects, (b) larger defects: Examples of mesh marking defects: (c) samples without grids; (d) the same samples as above, with grids to indicate mesh markings—the blue grid of 77 µm represents the size of the TiO_2_ screen mesh, and the green grid of 166 µm represents the size of the carbon screen mesh.

There are two main types of defect mechanisms evident. In Figure 7, examples of these defects are highlighted for better clarity into both the defect cause and the changes observed.

Figure 7a,b focus on defects which are inconsistent in distribution and size, varying from just a few microns detectable at the 40 µm resolution up to 100 µm in diameter. Frequently occurring in dusty samples and more evident at high dust levels it follows that these defects are resultant from the presence of dust particle contamination.

The small‐scale defects in Figure 7a show high PL but low LBIC, revealing an inverse relationship: better perovskite infiltration (high PL) occurs where the TiO_2_ layer is thinner, which reduces current flow (low LBIC) by creating a poor ETL–perovskite interface and so increasing recombination rates [30]. Higher recombination rates reduce V_oc_, as noted in JV curves of dusty devices (Figure S4). Additionally, blue‐ and red‐shifted PL peaks may indicate local variations in perovskite crystallization, caused by altered surface energy at dust particles or by the locally thinned TiO_2_ layer.

Larger defects (>50 µm) noted in Figure 7b show more complex luminescence patterns: a high‐PL, blue‐shifted center at the dust particle and a surrounding red‐shifted region. This suggests enhanced perovskite infiltration in areas where the TiO_2_ layer is thinned around the particle, increasing PL due to reduced injection into TiO_2_, while the larger surrounding area still has a thick enough TiO_2_ layer to maintain good interfacial contact and current flow (hence little change in LBIC). This indicates that TiO_2_ thinning is less significant and detrimental in larger defects. The central point corresponds to the non‐conductive dust particle.

Figure 7c focuses on a 500 µm area from each map of a dusty device, where consistently spaced bright spots are evident in PL intensity. Figure 7d adds an overlay of the mesh grids on the same defect images to highlight that these markings line up with screen mesh marks from the printing process. Interestingly, the mesh mark infiltration defects seen in the optical images are not the same as those evident in the luminescence maps. The markings of about 77 µm in the optical image correspond to the size and spacing of the TiO_2_ screen mesh [18] (blue grid), while those visible on the PP and LBIC maps are from the carbon screen mesh, with a spacing of about 166 µm (green grid).

TiO_2_ mesh marks are visible in the optical images, but they do not appear in any of the luminescence maps, likely due to the resolution of the PL and LBIC imaging and the reflective nature of TiO_2_ [24]. The carbon mesh marks are not visible on the optical image and are not therefore impeding perovskite infiltration lower in the stack. However, they do appear as bright spots in PL and correspond well with reduced LBIC, as well as occasional PP blue shifts, suggesting again that the high PL intensity coupled with poor LBIC at these points is indicative of poorer charge extraction [30], in this case at the carbon‐perovskite interface, while blue shifting could indicate some localized variation in perovskite crystal growth and suggests thinner, less conductive areas which allow effective perovskite infiltration but prevent efficient charge extraction [19, 25].

Dust‐related defects are most common and most visible when dust is present on the TiO_2_ layer, indicating that charge behavior is most affected when contamination occurs at the ETL/perovskite interface. Overall device performance remains largely unchanged, however, indicating that these defects do not lead to significant recombination or loss of charge carriers. Even so, dusty devices show slightly increased recombination losses, reflected in small changes in V_oc_ seen with all dusty samples. A reduction noted in J_sc_ in some dusty devices may have been caused by some dust particles blocking light attenuation on a very small scale, most apparent when dust was located on the TiO_2_ layer in moderately dusty devices, but as this did not follow for the same layer at higher dust levels despite evidence of increased defects here in the luminescence maps, it is likely this difference was due to batch variation. The most significant reduction in reproducibility noted in JV data when dust was located on the ZrO_2_ layer was linked to increasing the roughness of the carbon, as was evidenced in the WLI data. A small reduction was also noted in the FF in devices with dust at the ZrO_2_ layer suggests an increase in shunts.

### 2.3 Impact of Paste Contamination on Print Quality

The previous section analyzed the perspective of one mode of dust contamination to printed devices, with dust particles at printed layer interfaces. Contamination can also occur within pastes, which may affect print quality differently. Studies on the behavior and deposition of the pastes used to print m‐CPSCs have enabled careful optimization of particle, solvent and binder ratios to achieve the best quality prints possible [25, 31]. Contamination could easily impact the flow, spread and separation of such carefully balanced pastes, which could change the behavior of the pastes when printed. With the constant reuse of pastes, an environment with even slightly higher dust levels, this may significantly increase paste contamination levels, and this can add up over time. Dust contaminants could cause problems such as mesh blockages during printing, and contaminant particles may significantly impact ink rheology, which is known to influence print quality [31, 32]. Therefore, an additional set of experiments was designed to examine how dust contamination in the TiO_2_, ZrO_2,_ and carbon pastes used in this study may affect the quality of m‐CPSCs.

Rheological analyses were conducted to provide insight into how well the pastes will deposit during the screen‐printing process. The rheological profiles of the pastes determine how the ink will flow through the mesh and then separate, dictating the quantity of ink deposited as well as the topographical surface profile.

**FIGURE 8:**
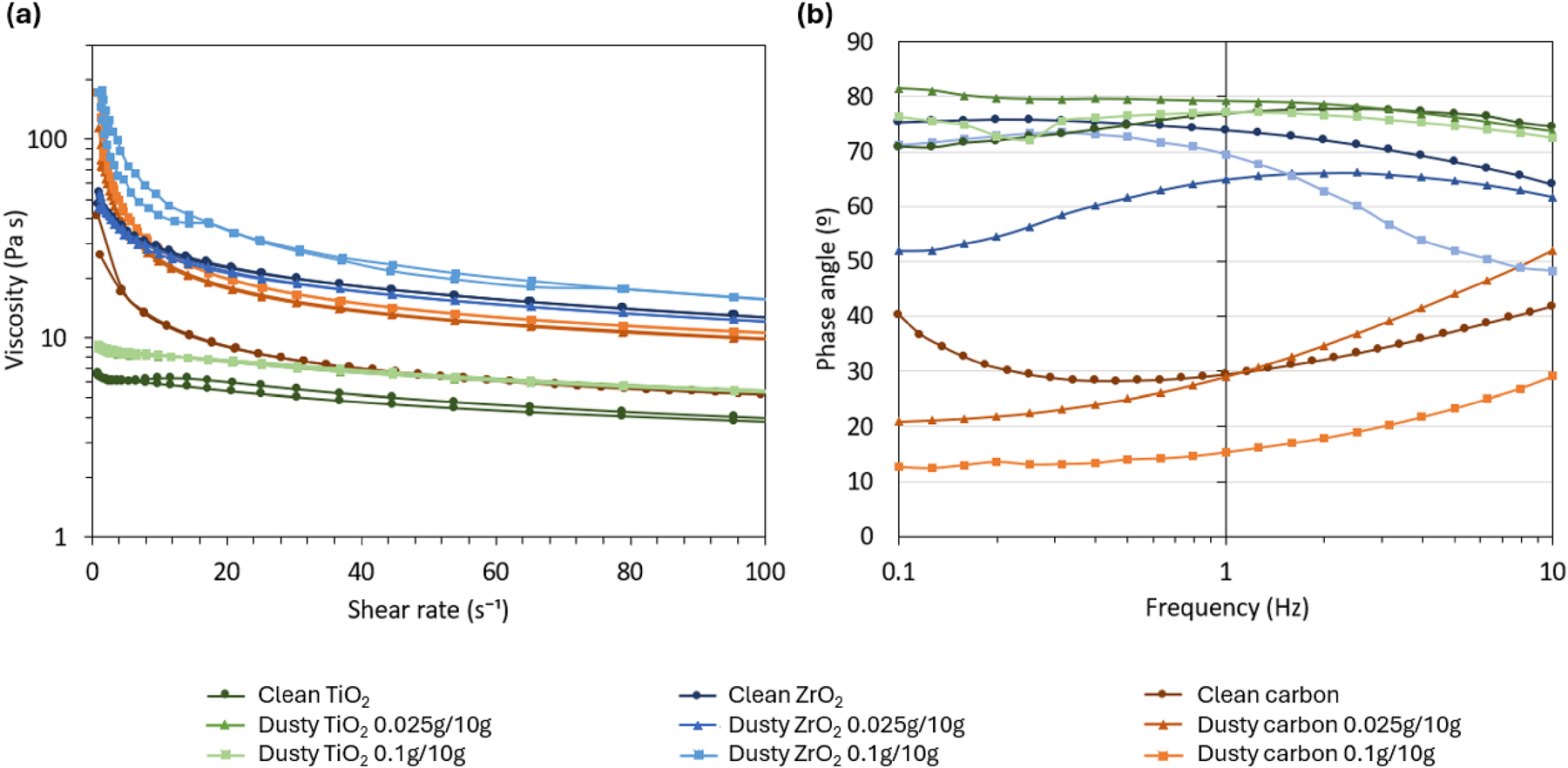
Rheology data showing (a) viscosity and (b) phase angle of TiO_2_, ZrO_2,_ and carbon pastes with 0.025 g of dust per 10 g, 0.1 g of dust per 10 g, and clean control samples.

To examine how paste contamination influenced the rheological profiles of the pastes, contaminated TiO_2_, ZrO_2,_ and carbon pastes were prepared with 0.025 g of dust per 10 g and 0.1 g of dust per 10 g, for comparison with uncontaminated pastes. The dust was mixed into the paste sample using a plastic spatula. Figure 8 shows charts of viscosity against shear rate (a), and phase angle against oscillation frequency (b) for these samples.

Dust contamination led to increases in viscosity in most pastes, which became more pronounced as contamination increased. Higher dust particle contamination will therefore likely have a more significant impact on print quality and decrease device performance to a greater extent. However, the extent of the observed rheological change was different in TiO_2_, ZrO_2,_ and carbon pastes.

The TiO_2_ paste exhibited the lowest viscosities out of the three pastes, and the least significant reduction in viscosity with increasing shear rate, indicating shear thinning (pseudoplastic) behaviors. When contaminants were added, there was negligible change in the viscosity profile with 0.025 g/10 g of dust, although there was a relatively small and even increase in viscosity across all shear rates with the larger concentration of dust (0.1 g/10 g). There was also a relatively small change in the elastic response, with elasticity remaining low, as indicated by the high phase angles between 70° and 80°. This may suggest that printed TiO_2_ films may have more tolerance to dust contamination in the paste, with a reduced likelihood of incoherent prints or mesh markings which cause inconsistent or rougher prints. The mesoporous TiO_2_ used here is an optimized dilution (1:0.75 dilution) of a commercially available paste [25].

The ZrO_2_ paste is a non‐diluted commercially available paste, exhibiting far higher viscosities across all shear rates and more significant pseudoplastic behavior than the TiO_2_ paste (Figure 8a). However, the viscoelastic profile (Figure 8b) for the uncontaminated ZrO_2_ paste displays a similar elasticity to the TiO_2_ paste. Dust contamination resulted in increased viscosity, but only at higher contamination of 0.1 g/10 g. Elasticity was impacted at lower particle concentrations, with the phase angle decreasing with dust addition. The ZrO_2_ sample with lower contamination of 0.025 g/10 g showed a larger increase. This discrepancy may be due to uneven contamination across the 10 g sample, resulting in more dust picked up for rheology testing. As with the TiO_2_, dust contamination of ZrO_2_ is likely to cause increased incidence of incomplete, incoherent prints and may be prone to more severe mesh marking.

The carbon paste is also a carefully optimized and diluted commercially available paste [19]. The clean sample shows a viscosity profile that lies between those of the TiO_2_ and ZrO_2_, and significantly higher elasticity. Adding dust contamination to the carbon paste had the largest impact on both elements of the paste's rheological profile, with significant increases in viscosity and elasticity. This means that increasing dust particle contamination will have a significant impact on print roughness and the occurrence of print defects such as mesh marks, agglomerates and pinholes. Carbon paste is likely more sensitive to contamination as the caron morphologies, concentrations, and ratio of carbon black to graphite have been optimized to promote mechanical stability and conductivity while maintaining flow [31]. This accounts for the much greater impact of particle contamination on viscosity in carbon paste.

Contaminated ZrO_2_ and carbon pastes are therefore likely to experience significant printing changes with particle contamination, while the less viscous TiO_2_ pastes are likely much more resilient towards contamination‐driven print changes.

**FIGURE 9:**
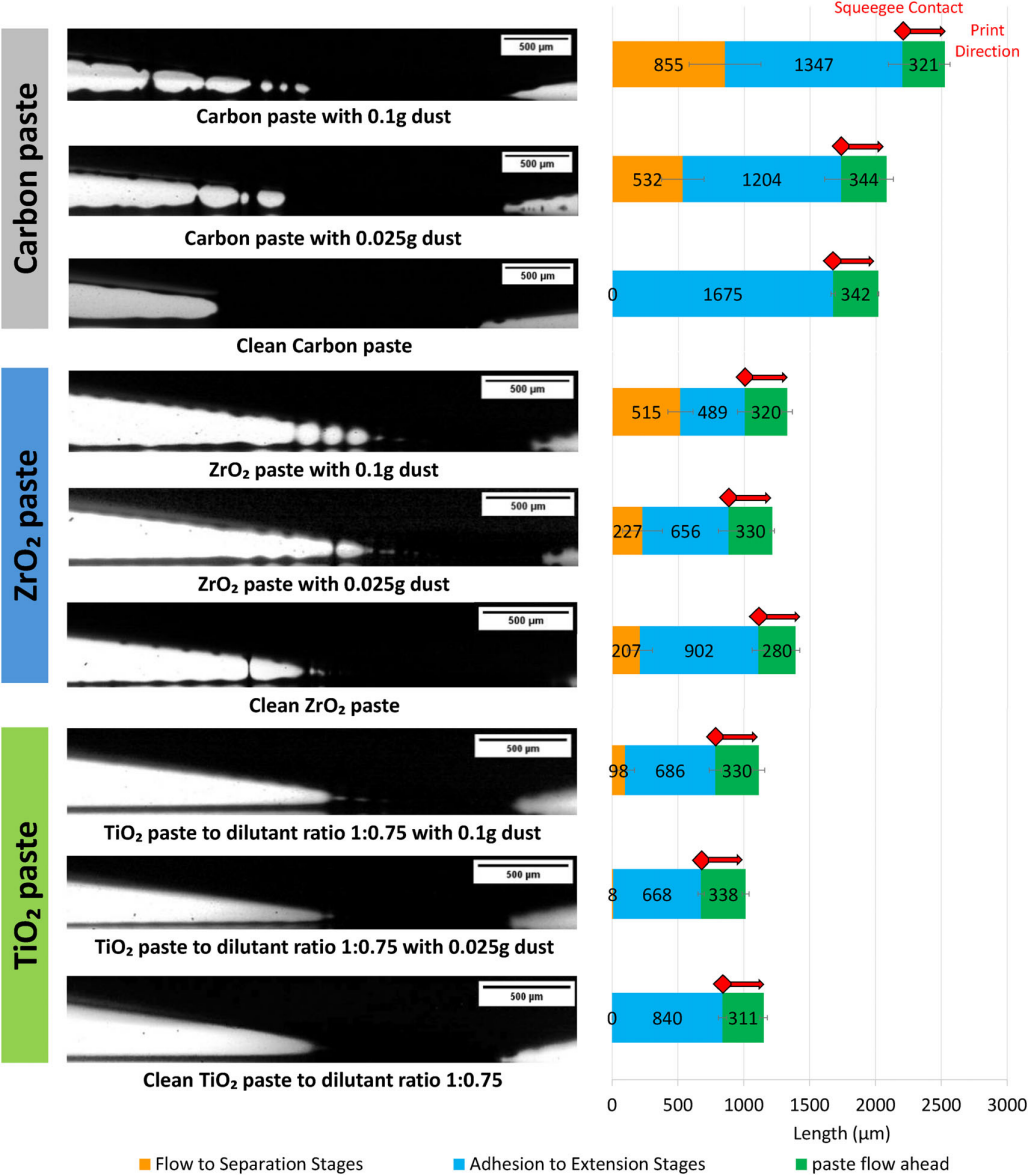
Screen print visualization (SPV) of clean versus dusty pastes: Carbon paste clean, 0.025 g dust/10 g paste and 0.1 g dust/10 g paste; ZrO_2_ paste clean, 0.025 g dust/10 g paste and 0.1 g dust/10 g paste; TiO_2_ paste optimized dilution ratio 1:0.75 clean, 0.025 g dust/10 g paste and 0.1 g dust/10 g paste.

To examine the specific impact of particle contamination on paste behavior during printing, screen print visualization (SPV) was performed (Figure 9). SPV is a new technique that provides a visual and quantified analysis of the ink separation mechanics during screen‐printing using a high‐speed camera, showing how changes in rheological behavior translate to print quality [32]. An ideal paste will consistently produce a smooth, non‐filamented separation with a long adhesion to the extension stage. This is important to produce a smooth, consistent and reproducible print, as filamenting pulls paste upwards has been proven to leave inconsistent peaks and mesh marks [31].

Clean and contaminated pastes were examined to check for changes in the occurrence of filaments and the length of each printing stage (Figure 9). An explanation of the descriptions and stages of screen print visualizations referred to in the results is shown in Figure S5.

As predicted by the rheological analysis, dust contamination showed a minimal impact on the TiO_2_ paste, with only a slight reduction in adhesion to the extension stage and only a single filament noted in the 0.025 g/10 g sample. The 0.1 g/10 g sample was slightly more impacted with a longer flow to the separation stage, but still produced a relatively clean separation with only two filaments. This led to minimal variation in the topographical profile of the deposited wet film, as shown to the left of the ink separation.

The ZrO_2_ paste was more affected, with a significant decrease in the adhesion to the extension stage, however the filamenting was similar to the clean ZrO_2_ sample. The carbon paste was impacted the most by 0.025 g of dust. The clean sample printed smoothly with no filamentation occurring with ink separation, whereas the 0.025 g sample showed a significantly reduced adhesion to extension stage and a long flow to the separation stage with notable filamentation. These impacts were then exacerbated with the addition of more contaminants in the 0.1 g samples, the detrimental effects being most evident in the ZrO_2_ and carbon samples with severe filamentation, leading to notable peaks and troughs in the deposited wet film, which would cause rougher prints.

Dust addition also introduced notable inconsistencies between print runs, as shown in Figure S6. Both print runs shown used the same contaminated paste sample but resulted in a significant difference in printing behavior. This resulted in adhesion to extension stages lengths that were both higher and lower than the clean sample, suggesting that 0.025 g of dust could potentially improve some devices while making others worse. Average adhesion to extension for a print run ranged between 140 and 400 µm, with anywhere between 1 and 6 filaments occurring within that region. These inconsistencies are likely due to natural variation of dust concentrations within the sample. While this many not necessarily hamper the peak device performance of a given batch, it may impact batch consistency which is important to consider when scaling module sizes and manufacturing rates.

These results align with the rheological data, showing the behavior of the TiO_2_ paste to be most resistant to dust contamination in the paste itself, and the carbon paste to be most impacted, as well as some inconsistencies depending on the concentration of dust particles within the paste. A low contamination dusty environment would therefore have an impact on the print quality of pastes that are higher viscosity and elasticity, such as ZrO_2_ and those with more finely balanced particle content such as carbon, increasing the difficulty of producing smooth, coherent and consistent prints. These impacts would be more apparent in higher contamination dusty environments, and therefore it is vital that screens and pastes are kept as clean as possible during print runs to avoid these problems as well as considering regular checks on pastes that are re‐used.

## 3 Conclusion

This work examines the impact of dust particle contamination in screen‐printed m‐CPSCs to assess the necessity of performing printing steps in a clean room environment. Two contamination routes were considered: interlayer dust contamination, where dust particles settle onto prints during drying, and paste contamination prior to printing the mesoporous layers.

Device performance showed good resilience to interlayer dust contamination, with devices achieving over 13% PCE even in samples with a high level of contamination. This was observed despite some interlayer contaminants reducing underlying m‐TiO_2_ infiltration and perovskite crystal quality in some cases, evidenced by changes in PL and LBIC around some particles. Particle contamination atop the TiO_2_ print had most impact on devices.

The main impact of interlayer contamination was to reduce reproducibility. This was attributed to increased print roughness‐ films printed onto contaminated layers were considerably rougher wit thin mesh marked areas surrounding underlying particles. Moderate dust levels affected J_sc_ consistency with FF, and V_oc_ also impacted at high dust levels. This loss of reproducibility occurred regardless of dust position within the stack, with the greatest impact observed in cells with dust at the ZrO_2_/carbon interface. Interlayer contaminants closer to the carbon layer produced greater roughness, further increasing PCE inconsistency.

The rheological behavior of TiO_2_, ZrO_2,_ and carbon pastes used for printing m‐CPSC devices was changed with the inclusion of dust particles. As well as introducing foreign particles to prints, contaminated pastes exhibited significantly increased viscosity and elasticity, increasing roughness and mesh marking in printed films. As the least viscous and elastic paste, the TiO_2_ was least affected by contamination with only minor changes eve n at high dust level. The largest effect was seen in the carbon paste, which requires a fine balance between carbon black and graphite for optimal printing.

The minimal impact of particle contamination on PCE suggests that it should be possible to manufacture high performing m‐CPSC devices outside of a cleanroom, significantly reducing the cost and maintenance requirements of potential laboratory or scale‐up facilities. The minor inconsistencies in performance should still be considered, and therefore a good level of cleanliness in printing areas is still important to consider. These results do highlight the importance of keeping dust particle contamination out of printing pastes in particular carbon, but that this could be mitigated by monitoring the rheology and utilizing SPV analysis of pastes that are regularly taken on and off printing screens for re‐use.

Further work in this area is required to consider the longer‐term effects of dust particles in m‐CPSCs, and whether this will influence device performance lifetime. Dust particles of other types are also worth considering as these may introduce other complexities to device behaviors.

This work highlights that m‐CPSCs and modules may be amenable to less stringent, lower‐cost, more scalable cleanliness controls, significantly enhancing their potential commercial viability compared to other architectures.

## 4 Materials and Methods

### 4.1 Materials Used

Titanium di‐isopropoxide bis(acetylacetonate) (TAA, 75% in IPA, Sigma‐Aldrich), anhydrous 2‐propanol (IPA, 99.5%, Sigma‐Aldrich), TiO_2_ paste (30NR‐D, GreatCell Solar)), ZrO_2_ paste (ZTSP, Solaronix), carbon paste (Gwent Electronic Materials), and terpineol (95%, Sigma‐Aldrich) were used as received. Precursor materials PbI_2_ (99%, Sigma‐Aldrich), MAI (CH_3_NH_3_I, anhydrous, GreatCell Solar), 5‐ammonium valeric acid iodide (5‐AVAI, GreatCell Solar), *γ*‐valerolactone (GVL, Sigma‐Aldrich), and anhydrous MeOH (99%, Sigma‐Aldrich) were used as received.

### 4.2 Screen Printing Device Fabrication

FTO substrates were patterned with a Nb/YVO4 laser (532 nm) before cleaning with ∼5% Hellmanex in deionized water, rinsing with acetone and IPA, and drying with N_2_. Substrates were then placed in a Nano plasma system (Diener Electronics), and plasma was cleaned for 8 mins in an O_2_ environment.

The substrate was heated to 300°C on a hot plate, and a compact TiO_2_ blocking layer was deposited by spray pyrolysis of 0.2 M titanium di‐isopropoxide‐bis(acetylacetonate) in IPA.

To form the mesoporous TiO_2_ layer, the titania paste (30NRD) was diluted 1:0.75 by weight in terpineol, screen printed and sintered at 500°C for 30 min after a slow ramp. Next, ZrO_2_ and carbon were printed and annealed at 400°C for 30 min each. All layers were printed and annealed in ambient conditions.

The AVA_0.03_MAPbI_3_ precursors were prepared by dissolving 0.0086 g 5‐AVAI, 0.1753 g MAI, and 0.5062 g PbI_2_ in a mixture of 0.9 mL GVL and 0.1 mL MeOH. All precursor solvent mixes were fabricated in an N_2_ glovebox to the specified concentration and stirred at room temperature until dissolved. Once fabricated, precursors were stored in dark ambient conditions (∼18°C and 30%–60% RH).

Devices were cooled to room temperature in ambient conditions (30%–50% RH, 18−21°C) before drop casting of 18–20 µL of room temperature precursor onto the stack surface. Devices were left for 30 min in ambient conditions after drop casting the precursor to ensure adequate infiltration before annealing on a hot plate for 1 h at 45°C unless otherwise stated. Contacts were applied with an ultrasonic solder at 180°C.

### 4.3 Characterization

The 1 cm^2^ active area was masked to 0.16 cm^2^ for testing. To ensure identical mask placement over multiple tests, tested areas (in the center of the active area) were marked prior to testing. A Keithley 2400 source meter and class AAA solar simulator (Newport Oriel Sol3A) at 1 sun were used for JV measurements (calibrated against a KG5 filtered silicon reference cell, Newport Oriel 91150‐KG5). Devices were scanned at a rate of 100 mV/s from 1.1 to −0.2 V and vice versa after a light soaking period of 180 s.

Optical microscopy was carried out using a Zeiss Primotech optical microscope at 10× zoom.

SEM images were obtained using a JEOL JSL 7800F FEG scanning electron microscope (SEM). Cross‐section samples were broken across the middle of the device using a glass scribe, then mounted onto a hexagonal nut to face them upwards. All samples were sputter coated in 8 nm of platinum for improved conductivity. Samples were imaged using 15 kV of electron energy and images were captured at varying magnifications. Chemical analyses were carried out with an Oxford Instruments Ultim energy‐dispersive x‐ray spectroscopy (EDS) detector with AZTEC software (v6.0) analysis package at a 10 mm working distance.

White light interferometry (NT9300, Veeco Instruments, Inc., Plainview, NY, USA) was used to measure a full 3D surface profile of the dry, printed layers with the specified routine: 5× magnification was used, giving a measurement area of 1.2 by 0.93 mm (at a resolution of 736 × 480 pixels with sampling at 1.67 µm intervals). Average surface roughness measurements (S_a_ and S_z_) over the printed area were taken from the middle of each print. A total of nine measurements were taken for each setting, from which the average and standard deviation were calculated.

PL mapping was carried out using a Renishaw InVia confocal Raman microscope. Device areas measured were 4000 µm × 4000 µm with a step size of 40 µm. The emission wavelength for all samples was measured at 775 nm. PL, PP, and LBIC map data is acquired simultaneously in one scan at short circuit. To acquire the photocurrent, the device electrodes were connected to a lock‐in amplifier (Stanford Research SR830) with a chopper cutting the laser beam at 75 Hz.

Rheological evaluation was carried out using a combination of shear and viscoelastic measurements. Shear viscosity measurements were carried out on a Malvern Bohlin rotational rheometer (Gemini Bohlin Nano, Malvern Instruments, Malvern Panalytical Ltd, Malvern, UK) with a 2° 20 mm stainless steel cone and a parallel plate held at 25°C. Ink viscosity was measured as the shear rate was gradually increased to 100s^−1^ and then reduced back to 1s^−1^. Viscoelastic measurements were carried out on a Malvern Kinexus Pro Rheometer (Malvern Instruments, Malvern Panalytical Ltd., Malvern, UK) with a 1° 50 mm stainless steel cone and a parallel plate. Amplitude (strain) sweep measurements were conducted to establish the linear viscoelastic range at 0.1, 1, and 10 Hz. Then, using a stress within the established linear viscoelastic region, a frequency sweep from 0.1:10 Hz was conducted.

#### 4.3.1 Screen Printing Visualization

The mesh‐substrate ink separation occurring during screen‐printing was captured on a high‐speed camera (Photron FastCam Mini High‐Speed Camera) at a frame rate of 125 frames per second, with 8× magnification, and a 10 000‐lux lamp used for backlighting. The printing was conducted on a custom‐made screen‐printing apparatus, the screen‐printing Visualizer (SPV). A polyester mesh at 22.5° with 130 threads per cm, 34 µm thread diameter, and 9‐micron emulsion was used to print the image. A 65–70 Shore A hardness diamond squeegee was used, along with a snap distance (distance between screen and substrate) of 1.825 mm and squeegee travel speed of 300 cm min^−1^ (50 mm s^−1^). The substrate was PET (polyethylene terephthalate—Melinex 339, DuPont Teijin Films (175 µm thickness) opaque white). The print image consisted of a continuous 75 µm wide line in the direction of squeegee travel. The ink separation was quantified with two key regions, the adhesion to extension region (where the ink remains in continuous contact with the mesh and substrate), and the flow to separation region (where the main body of ink splits off into filaments that eventually separate). A total of 45 sets of measurements were taken for each ink (15 sets of measurements conducted for each of the three prints, across evenly spaced intervals in the region assessed with high‐speed imaging) from which the average and standard deviation in each of the print stages for each dilution was calculated.

## Conflicts of Interest

The authors declare no conflicts of interest.

## Supporting information




**Supporting File**: smll73299‐sup‐0001‐SuppMat.docx.

## Data Availability

The data that support the findings of this study are available from the corresponding author upon reasonable request.
