# Supplementary material for: The Impact of Dust Particles on the Function of Screen‐Printed Triple‐Mesoscopic Perovskite Solar Cells
Source: Small. 2026 Apr 7;22(29):e73299. doi: 10.1002/smll.73299 (PMC13206305; doi:10.1002/smll.73299)
Supplement: Supplementary file 1 — Supporting File: smll73299‐sup‐0001‐SuppMat.docx. [file SMLL-22-e73299-s001.docx]

***The impact of dust particles on the function of screen printed triple mesoscopic perovskite solar cells***

*Kathryn Lacey^1^, Sarah-Jane Dunlop-Potts^1^, Carys Worsley^1^, Rodrigo Garcia-Rodriguez^1^, Tom Dunlop^2^, Declan Hughes^1^, Krishna Seunarine^1^, Matthew Davies^3^, Trystan Watson^1^**

^1^ SPECIFIC IKC, Faculty of Science & Engineering, Swansea University, Fabian Way, Swansea, SA1 8EN, UK.

^2^AIM, Faculty of Science & Engineering, Swansea University, Fabian Way, Swansea, SA1 8EN, UK.

^3^School of Chemistry and Physics, University of KwaZulu-Natal, Durban, South Africa.

*[t.m.watson@swansea.ac.uk](mailto:t.m.watson@swansea.ac.uk)

***Supplementary figures***


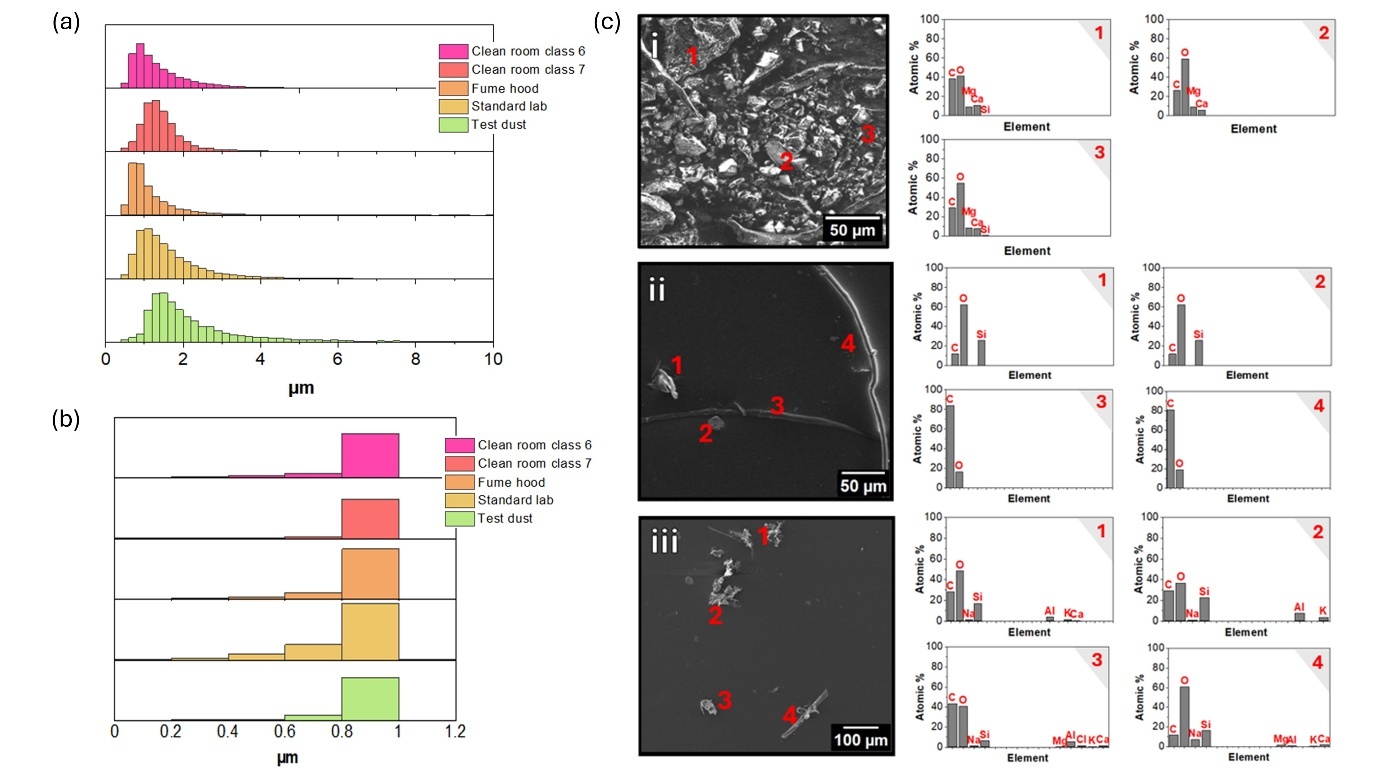


**Supplementary figure S1:** **Dust particle size, morphology and chemistry characterisation**; (a) particle size analysis for varying laboratory environments; (b) particle roundness analysis for varying laboratory environments; (c) SEM images and EDS results from (i) sample of test dust, (ii) standard laboratory, and (iii) ISO class 7 cleanroom.


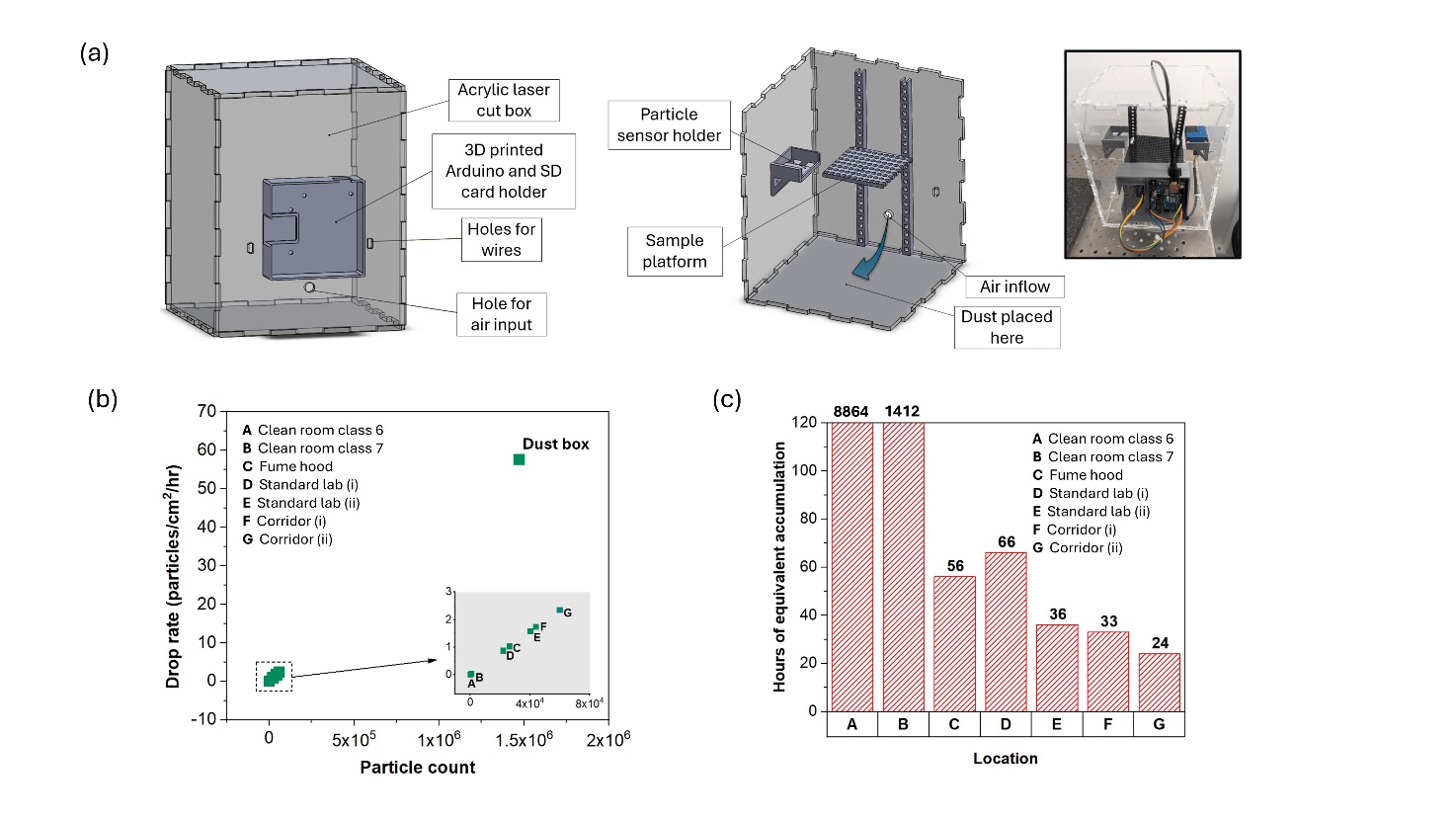


**Supplementary figure S2:** **Design, setup and context of dust box experiment:** (a) outside and inside schematics of dust box alongside photograph of final dust box setup; (b) scatter graph of particle drop-out rate vs. particle count in different areas compared with dust box; (c) bar graph of the hours of dust particle accumulation the dust box circulation is equivalent to in each area.


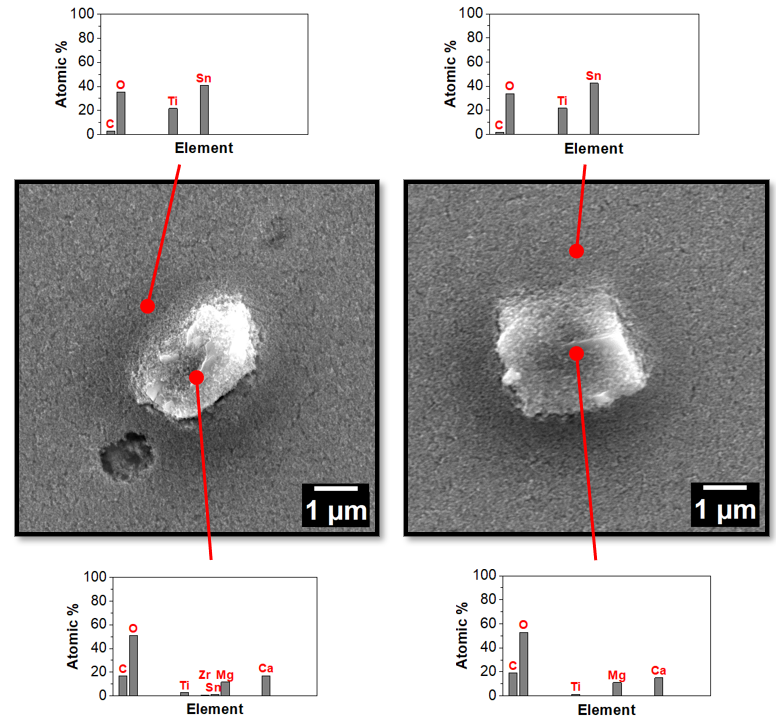


**Supplementary figure S3**: SEM images and EDS analysis of individual dust particles within annealed TiO_2_ print.


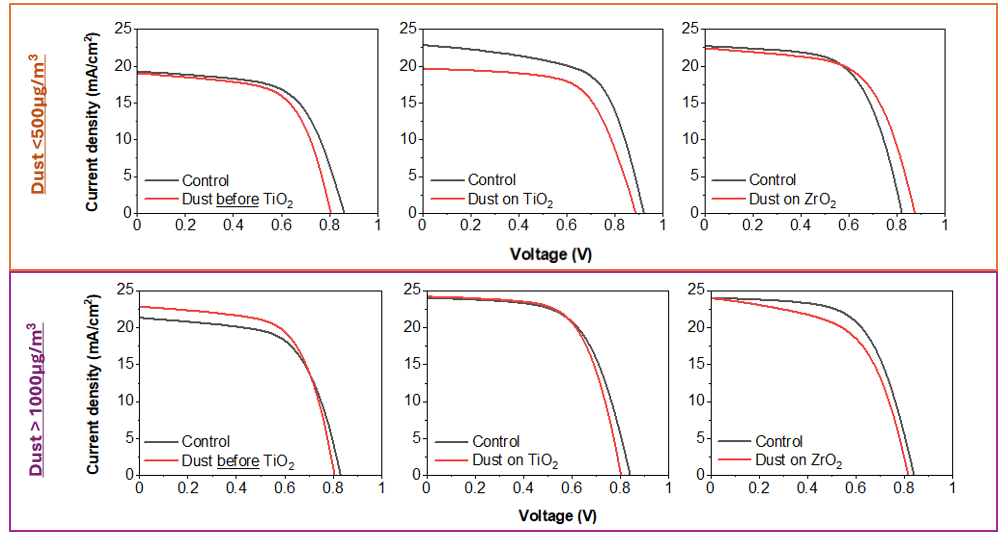


**Supplementary figure S4**: JV curves of hero devices in each batch (dusty vs. control samples)


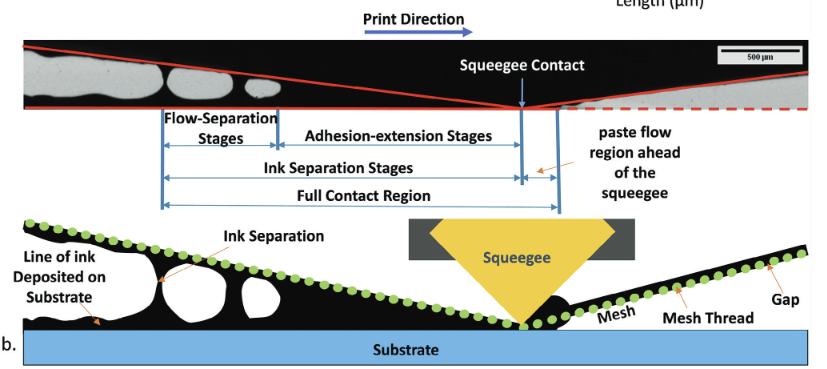


**Supplementary figure S5**: Explanation of SPV methods


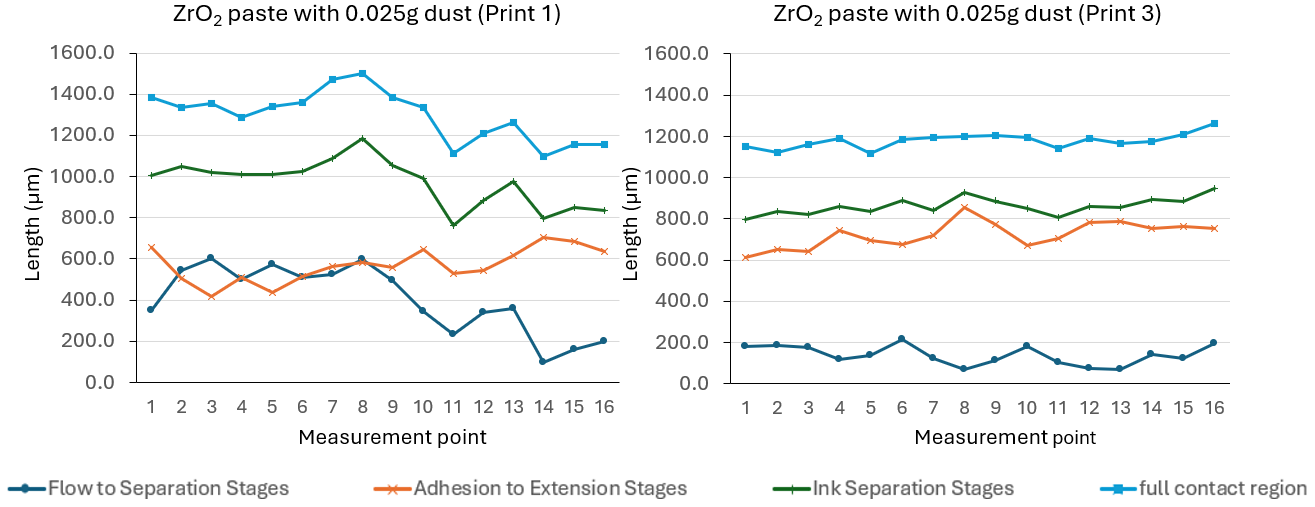


**Supplementary figure S6**: Repeats of ZrO_2_ rheology showing inconsistency in paste behaviours.
